# Taxonomic Revision of *Solorina* (Peltigeraceae, Ascomycota), Reveals a New Genus and Three New Species

**DOI:** 10.3390/jof11030169

**Published:** 2025-02-20

**Authors:** Ting Zheng, Lisong Wang, Min Ai, Yuxin Gan, Rong Fan, Yingjun Zhang, Fiona Ruth Worthy, Jizhen Jin, Wenping Meng, Shengbang Zhang, Xinyu Wang

**Affiliations:** 1 State Key Laboratory of Phytochemistry and Natural Medicines, Kunming Institute of Botany, Chinese Academy of Sciences, Kunming 650201, China; zhengtinggz@163.com (T.Z.); wanglisong@mail.kib.ac.cn (L.W.); aimin@mail.kib.ac.cn (M.A.); ganyuxin@mail.kib.ac.cn (Y.G.); fanrong@mail.kib.ac.cn (R.F.); zhangyingjun@mail.kib.ac.cn (Y.Z.); fiona.worthy@outlook.com (F.R.W.); jinjizhen1@outlook.com (J.J.); 2 Guizhou Botanical Garden, Guiyang 550004, China; mengwp123@163.com; 3 Key Laboratory for Biodiversity Conservation in Karst Mountain Area of Southwestern China of the National Forestry and Grass Administration, Guiyang 550004, China; 4 Yunnan Key Laboratory for Fungal Diversity and Green Development, Kunming Institute of Botany, Chinese Academy of Sciences, Kunming 650201, China; 5 Key Laboratory of Plant Stress Research, College of Life Sciences, Shandong Normal University, Jinan 250014, China; 6 Qinghai Shanshui Natural Resources Survey Institute, Xining 810008, China; 18909710218@163.com

**Keywords:** genus delimitation, *Pseudosolorina*, lichen, phylogeny, taxonomy

## Abstract

The lichen genus *Solorina* exhibits significant morphological and chemical variations between species. Recent molecular studies have demonstrated that *Solorina* is polyphyletic, underscoring the need for a comprehensive taxonomic revision. Phylogenetic analyses employing Bayesian methods and Maximum Likelihood approaches based on three molecular loci (nrITS, nrLSU, mtSSU) revealed that species of *Solorina* segregate into two distinct clades. The first clade includes species characterized by bright orange lower surfaces that contain secondary metabolites, notably solorinic acid. The type species, *Solorina crocea*, is retained in the genus *Solorina*. The second clade encompasses species with white or brownish lower surfaces; most species lack secondary metabolites and are now classified as a new genus, *Pseudosolorina*. As a result of this taxonomic revision, two species: *S. crocea* and *S. crocoides* remain in the genus *Solorina*. Five species with white or brownish lower surfaces were transferred to the new genus *Pseudosolorina*, which consists of three newly described species and five new combinations. Four species previously described as *Solorina: S. embolina*, *S. fuegiensis*, *S. octospora*, and *S. platycarpa* have morphology consistent with *Pseudosolorina*, but are currently retained in *Solorina* due to the absence of supporting DNA sequence data. A key to *Solorina* and *Pseudosolorina* is provided. The spores of *S. crocea* exhibit wall ornamentation featuring rounded papillae, which are distinct from those of *Pseudosolorina*. Molecular data and morphological characters also indicate that both *Solorina* and *Pseudosolorina* engage in symbiotic associations with photobionts cyanobacteria *Nostoc* and chlorophytes *Coccomyxa* or *Asterochloris*.

## 1. Introduction

The lichen genus *Solorina* Ach. belongs to Peltigeraceae, Peltigerales, Lecanoromycetidae, Lecanoromycetes, Ascomycota [1,2]. Since Linné first reported the lichen species *Lichen croceus* L. and *Lichen saccatus* L. in the 1750s [3,4], the taxonomic placement of these species has garnered significant interest among lichenologists. After several modifications, Acharius established the new genus *Solorina* Ach. to accommodate these two species, with *S. crocea* (L.) Ach. designated as the type species. The genus *Solorina* is characterized by its fragile foliose thallus, paraplectenchymatous upper cortex, and large, rounded apothecia that are immersed in the upper surface, without a margin, typically exhibiting a concave disc that ranges from dark red-brown to nearly black [1]. Later lichenologists, including Nylander and Gyelnik, assigned additional species to this genus [5,6,7,8,9,10,11,12]. Currently, *Solorina* comprises approximately ten species [13], most of which inhabit calcareous soils in high-altitude mountainous regions and exhibit a bipolar distribution. Six species and one variant have been reported from China [14].

Species of *Solorina* exhibit considerable variation in thallus morphology and the composition of their photobiont layers. Differences in these photobiont layers have been considered a taxonomically distinctive trait. Nylander described the genus *Solorinina* Nyl. as containing a cyanobacterial photobiont layer, with *Solorina* possessing a chlorophyte photobiont layer [15]; Hue further classified *Solorina* into three sections, *Pleurothea* Hue with two separate photobiont layers, *Solorina* accommodating three species with a single cyanobacterial photobiont layer, and *Eusolorina* Hue accommodating species featuring a single chlorophyte photobiont layer [12]. Gyelnik reclassified *Solorina* into two subgenera: *Eusolorina* Hue and *Solorinina* (Nyl.) Hue, and further divided them into four sections (*Protosolorina* Gyeln., *Neosolorina* Gyeln., *Protosolorinina* Gyeln., and *Neosolorinina* Gyeln.), based on variations in photobiont layers and the size of thallus [16]. Räsänen proposed the section *Neosolorina* (Gyeln.) Räsänen as a new genus [17]. However, these classifications are no longer widely recognized.

Currently, the species of *Solorina* are primarily distinguished based on the morphology of their thallus and ascospores. Ascospore numbers differ between species, with formations including 1, 2, 4, and 8 spores per ascus; the spores are thick-walled and appear roughened under light microscopy. Some studies classify species of *Solorina* based on the number and ornamentation of the spores [18,19,20]. Since most *Solorina* species lack secondary metabolites, only four species have been distinguished based on their chemical characteristics, notably *S. crocea*, which contains numerous secondary metabolites, including solorinic acid and norsolorinic acid [21,22]. The morphology of *S. crocea* and *S. crocoides* (Nyl.) Gyeln. differ significantly from other *Solorina* species by the bright orange color of their lower surface.

Recent molecular studies indicated that *S. crocea* diverged from all other species of *Solorina* [2,23]. DNA data were lacking, and records were scarce for *S. crocoides*. Because *S. crocoides* was initially reported from the Himalayas, for the purposes of this study we collected new samples from Xizang, China, from which DNA sequences were obtained. We also collected a further 36 specimens of other *Solorina* spp., from which we obtained DNA sequences. The phylogenetic result confirms that *Solorina* is polyphyletic and introduces a new genus, *Pseudosolorina* T. Zheng and Li S. Wang.

## 2. Materials and Methods

### 2.1. Morphological, Anatomical and Chemical Characters

Most materials for this study were collected in China, and specimens were stored in the Herbarium, Kunming Institute of Botany, Chinese Academy of Sciences (KUN). Type specimens of *S. embolina*, *S. octospora*, and *S. simensis* were borrowed from the University of Helsinki (H). High-resolution photographs of type or lectotype specimens of *S. crocea*, *S. bispora*, *S. fuegiensis*, and *S. saccata* were provided by the curators of Linnaean Herbarium (LINN) or obtained from the website of Global Plants (https://plants.jstor.org/, accessed on 12 September 2023).

The specimens were examined using standard microscopy techniques. Morphological descriptions were based on observations using a Nikon SMZ 745T (Minato City, Japan) dissecting microscope and a Zeiss Axio Scope A1 stereomicroscope (Oberkochen, Germany). Scanning electron microscopy ZEISS SIGMA 300 (Oberkochen, Germany) was conducted on selected specimens to visualize spore ornamentation. The identification of secondary metabolites was performed using thin-layer chromatography (TLC) [24,25] and gradient-elution high-performance liquid chromatography (HPLC) [26].

### 2.2. DNA Extraction, PCR Amplification and Sequencing

Genomic DNA was extracted from dry or fresh specimens following the manufacturers’ instructions using the DNA secure Plant Kit (Tiangen, Beijing, China). Three gene loci of *Solorina* were amplified using the following primers: ITS1F [27], ITS4 [28], LR0R [29], LR5 [30], mrSSU1 and mrSSU3R [31]. The 16S rRNA genes of cyanobacteria were amplified with primers CYA359F and CYA781R-a [32]. The nrITS genes of the chlorophytes were amplified with primers nr-SSU-1780-5′ and ITS4T [33,34]. The 25 μL PCR mixture consisted of 2 μL DNA template, 1 μL of each primer, 12.5 μL 2 × Taq PCR MasterMix (Aidlab, Hong Kong) (Taq DNA Polymerase [0.1 unit/mL]; 4 mM MgCl_2_; and 0.4 mM dNTPs) and 8.5 μL ddH_2_O. The PCR settings and primer profile followed Zhao et al. [35]. Polymerase chain reaction (PCR) products were sequenced by Sangon Biotech (Shanghai, China).

### 2.3. Molecular Phylogenetic Analyses

The raw sequences were assembled and edited using SeqMan v.7.0 (DNAstar packages). Sequences extracted from new materials corresponding to each gene locus were aligned with additional sequences available from GenBank (Table 1) using the Concatenate Sequence feature and MAFFT v.7.505 [36] to generate nrITS-nrLSU-mtSSU matrices. The concatenated alignments were estimated by PartitionFinder 2.1.1 [37], based on the Bayesian Information Criterion (BIC), to find the most appropriate nucleotide substitution model for each of the three loci Phylogenetic relationships were inferred using Bayesian Inference (BI) and Maximum Likelihood (ML) methods, with the genus *Pannaria* as the outgroup. ML analyses were performed with IQ-TREE v2.2.0 [38] using the model SYM + I + G for nrITS, GTR + G for mtSSU and nrLSU. The Bayesian method was performed with MrBayes v.3.2.7 [39]. Four Markov chains were run with 2 million generations for each dataset, and trees were sampled every 100 generations. Convergence was assessed by ensuring that the average standard deviation of split frequencies was less than 0.01. Posterior probabilities greater than 0.9 and bootstrap support values above 80% were considered to provide significant support for the phylogenetic relationships. All the trees were visualized using FigTree v. 1.4.0 [40]. The identity of cyanobacteria and chlorophyte sequences were determined through BLAST analysis on the NCBI online server (https://blast.ncbi.nlm.nih.gov/Blast.cgi, accessed on 22 November 2024).

## 3. Results

### 3.1. Phylogenetic Analysis

**Figure 1:**
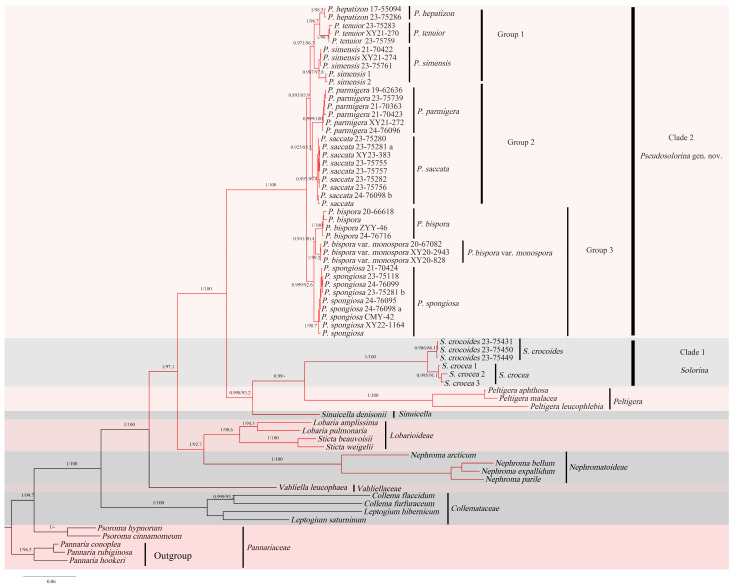
Phylogenetic relationships within Peltigerales were assessed using Bayesian Inference (BI), analyzing combined sequence data from nrITS, nrLSU, and mtSSU. Nodes supported by Bayesian posterior probability/ML bootstrap values ≥ 0.80/80% are denoted in the results. Maximum Likelihood bootstrap values and posterior probabilities are displayed proximal to the respective nodes. The red branches represent Peltigeraceae.

In this study, we generated 92 new sequences from 39 specimens and downloaded 78 additional sequences from GenBank; the final alignment for the combined regions dataset (620 bp for nuITS sequences; 552 bp for nrLSU sequences; 605 bp for mtSSU sequences) comprised 66 terminals (Table 1). Results from phylogenetic analyses presented here clearly indicate that the species of *Solorina* were divided into two well-supported clades: clade 1 and clade 2 (Figure 1). Clade 1 contains the type species of *Solorina*, *S. crocea*, so we retain this clade as the revised genus *Solorina*. The revised *Solorina* clade contains two species *S. crocea* and *S. crocoides*; these both have a bright orange lower surface, which is distinctly veined.

All species in clade 2, are characterized by a white or brownish lower surface. Clade 2 forms a monophyletic group with strong support for its division from clade 1. Consequently, we propose a new genus, *Pseudosolorina*, to classify this clade. Here, we present three new species and five new combinations for the new genus *Pseudosolorina*. We further subdivide the genus *Pseudosolorina* into three groups. The first group contains three species, *P. hepatizon*, *P. simensis*, and *P. tenuior*, which share thallus and apothecium morphological characters and have a single photobiont layer containing both cyanobacteria and chlorophytes. Group 2 contains two species, *P. parmigera* and *P. saccata*, with a green, chlorophyte photobiont layer and thallus over 2 cm in diam. Group 3 contains three species, *P. bispora*, *P. bispora* var. *monospora*, and *P. spongiosa*, with a green, chlorophyte photobiont layer and thallus less than 2 cm in diam. The smallest thallus diameter is for *P. spongiosa*, which is reduced to a circle around the apothecium.

### 3.2. Chemical Analysis

*Solorina crocea* contained several secondary metabolites, including solorinic acid, averantin, 6-*O*-methylaverantin, and gyrophoric acid. *S. crocoides* shared the same chemical constituents as *S. crocea*, except for the absence of gyrophoric acid. In the genus *Pseudosolorina*, the species *P. tenuior* and *P. simensis* contained gyrophoric acid, methyl gyrophorate, tenuiorin and 2′-*O*-methyltenuiorin. Other species in the genus: *P. bispora*, *P. bispora* var. *monospora*, *P. hepatizon*, *P. parmigera*, *P. saccata* and *P. spongiosa* lacked secondary metabolites.

### 3.3. Scanning Electron Microscopy for Spore Ornamentation

**Figure 2:**
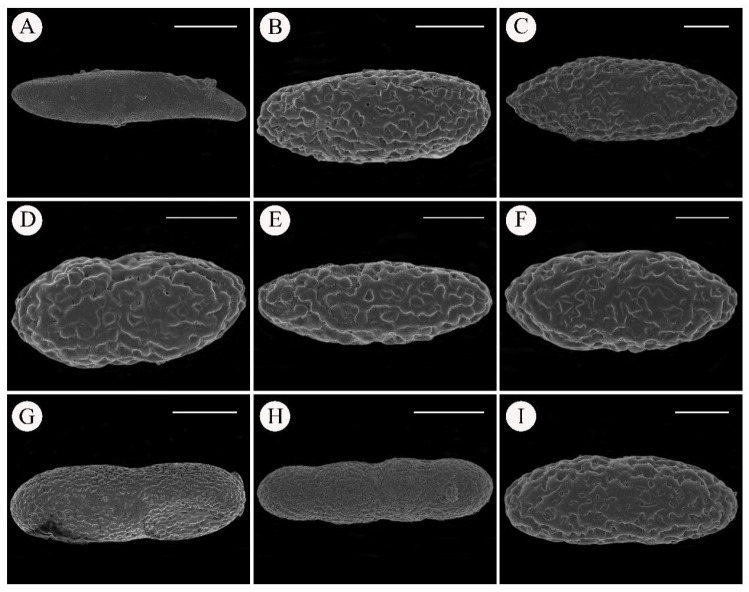
Scanning electron microscopy pictures of the spore of the *Solorina* and *Pseudosolorina* species. (**A**), *S. crocea* (Theodore and Esslinger 11831 KUN); (**B**), *P*. *hepatizon* (L. S. Wang et al. 23-75286 KUN); (**C**), *P*. *tenuior* (L. S. Wang et al. 23-75283 KUN); (**D**), *P*. *simensis* (X. Y. Wang et al. 21-70422 KUN); (**E**), *P*. *parmigera* (X. Y. Wang et al. 21-70423 KUN); (**F**), *P. saccata* (L. S. Wang et al. 23-75757 KUN); (**G**), *P. bispora* (Y. Y. Zhang ZYY-46 KUN); (**H**), *P. bispora* var. *monospora* (L. S. Wang et al. 20-67082 KUN); (**I**), *P. spongiosa* (L. S. Wang et al. 23-75119 KUN). Scale bars: 10 µm (**A**–**F**,**I**); 30 µm (**G**,**H**).

Analysis of spores by SEM (Figure 2) showed that *Solorina crocea* has a relatively smooth spore surface characterized by nipple-like ornamentations (Figure 2A). Conversely, the species of *Pseudosolorina* have a rough spore surface adorned with generally broad reticulating ridges and irregular lacunae ornamentations (Figure 2B–I). Although there are some differences in ornamentations among the various species of *Pseudosolorina*, distinguishing them solely based on this character would be challenging. No apothecia have been observed for *S. crocoides*, so no images are presented for the spores of this species.

### 3.4. Composition of the Photobionts

Molecular evidence suggests that *Solorina* and *Pseudosolorina* share photobionts, comprising cyanobacteria *Nostoc* and chlorophytes *Coccomyxa* or *Asterochloris* (Table 2). Morphological examinations of specimens of *S. crocea* reveal the presence of two distinct layers of photobionts: an upper bright green layer and a discontinuous blue lower layer. Previous reports indicated that *S. crocea* also contains the photobionts *Coccomyxa* and *Nostoc* [41], suggesting that they have an upper layer of *Coccomyxa* and a lower layer of *Nostoc*. In contrast, specimens from the species *S. crocoides*, exhibited only a single photobiont layer with both blue and bright green cells and did not possess cephalodia. Molecular data showed that these photobionts were *Nostoc* and *Asterochloris*, indicating that *S. crocoides* contains both cyanobionts and chlorobionts within the same photobiont layer. Within *Pseudosolorina*: *P. hepatizon*, *P. simensis*, and *P. tenuior* share characteristics of the photobiont layer found for *S. crocoides*: they contain *Nostoc* and *Asterochloris* within the same single photobiont layer. In contrast, *P. bispora*, *P. bispora* var. *monospora*, *P. parmigera*, *P. saccata* and *P. spongiosa* contain *Nostoc* and *Coccomyxa*, but with a single green photobiont layer only contain *Coccomyxa*, and *Nostoc* present only in cephalodia.

### 3.5. Revised Boundary for the Genus Solorina 

The genus *Solorina* has traditionally been recognized by its large, rounded, impressed apothecia that are immersed in the upper surface and typically concave, without a thalline margin. These traits distinguish *Solorina* from its sister groups, *Peltigera* Willd. and *Sinuicella* D. F. Stone, McCune & Miądl. However, significant morphological and chemical variations exist among the species within this genus. Furthermore, molecular studies suggest that *Solorina* is polyphyletic [2,23], indicating that these traditional characteristics are inadequate for reliably distinguishing this genus.

Our three-loci-based analysis confirms previous findings regarding the polyphyly of *Solorina* [2,23], revealing the division of this genus into two distinct clades. Based on the phylogenetic analyses and morphological and chemical characters, we define these two clades as representing two genera. The clade with a bright orange lower surface, which includes the type species *S. crocea*, was designated as the revised *Solorina*. According to this re-circumscription, species within *Solorina* are characterized by a bright orange lower surface and the presence of distinctive secondary metabolites, including solorinic acid. These species have apothecia which are flat or convexly immersed in the upper surface of the thallus (or apothecia absent), spores with nipple-like ornamentations, photobiont layer containing both cyanobacteria *Nostoc* and chlorophyte *Coccomyxa* or *Asterochloris*, secondary metabolites including solorinic acid, averantin, 6-*O*-methylaverantin, and gyrophoric acid. The combination of these secondary metabolites produces the characteristic orange lower surface.

Within the second well-defined monophyletic clade, all species have white or brownish lower thallus surfaces. This clade was established as a new genus *Pseudosolorina* to accommodate the species which were excluded from the revised definition of *Solorina*. The species within *Pseudosolorina* all exhibit apothecia immersed in the upper surface and slightly to deeply concave; their spores are distinguished by having broad reticulating ridges and irregular lacunae ornamentations. The photobiont layer contains *Nostoc* and *Asterochloris* or solely *Coccomyxa*, with *Nostoc* confined to cephalodia. Most species in *Pseudosolorina* lack secondary metabolites. This taxonomic revision provides new classification boundaries for the genus *Solorina* and *Pseudosolorina*, based on morphological, phylogenetic, and chemical characteristics.

There are four other species previously defined as *Solorina*, for which genetic material was unavailable. Of these, *S. octospora* Arnold and *S. platycarpa* Hue had been reported from China, whereas *S. embolina* Nyl. and *S. fuegiensis* C.W. Dodge were reported from elsewhere. These four species have white or brownish lower surfaces; thus, morphologically, they could be assigned to the new genus *Pseudosolorina*. However, due to the absence of sequence data, they must currently be retained within *Solorina*. Further research is needed to clarify the phylogenetic position of these species.

### 3.6. Taxonomy

***Solorina*** Ach., *K. Vetensk-Acad. Nya Handl.* 29: 228 (1808)

Type species: *Solorina crocea* (L.) Ach., K. Vetensk-Acad. Nya Handl. 29: 228 (1808)

Description: Thallus: large foliose, fragile, wide-spreading, dorsiventral, heteromerous, lobate; Lobes: rounded, margins raised; Upper surface: olive-green to green-gray when wet, red-brown to gray-brown when dry, smooth to scabrid, one species with soredia; Upper cortex: paraplectenchymatous; Photobiont: *Coccomyxa* and *Nostoc*; Lower cortex: distinct lower cortex lacking; Lower surface: bright orange, distinctly brownish veined, tomentose, with clusters of simple or branched rhizines; Apothecia: present or absent, large, rounded, irregularly scattered, not or only a little depressed into the thallus, flat or convex, thalline margin absent; Asci: clavate, *Peltigera*-type, 8-spored; Ascospores: red-brown or dark brown, 1-septate with a median constriction, ellipsoid to fusiform, wall surface ornamented with rounded papillae.

Chemistry: Solorinic acid, averantin, 6-*O*-methylaverantin and gyrophoric acid (detected by TLC and HPLC).

Ecology: Distributed in high-altitude areas and mainly grows on calcareous soil.

Note: Species with apothecia immersed into the upper surface and without margins were previously included in *Solorina*. However, a three-loci phylogenetic analysis confirmed that this genus was polyphyletic. The species with white or brown lower surfaces were excluded from *Solorina* to establish a new genus named *Pseudosolorina*. *Solorina* is now characterized by its immersed apothecia, bright orange lower surface, rounded papillate ornamentation of the spores, and distinctive secondary metabolites, notably solorinic acid. Members of this genus are typically found in alpine habitats.

**Figure 3:**
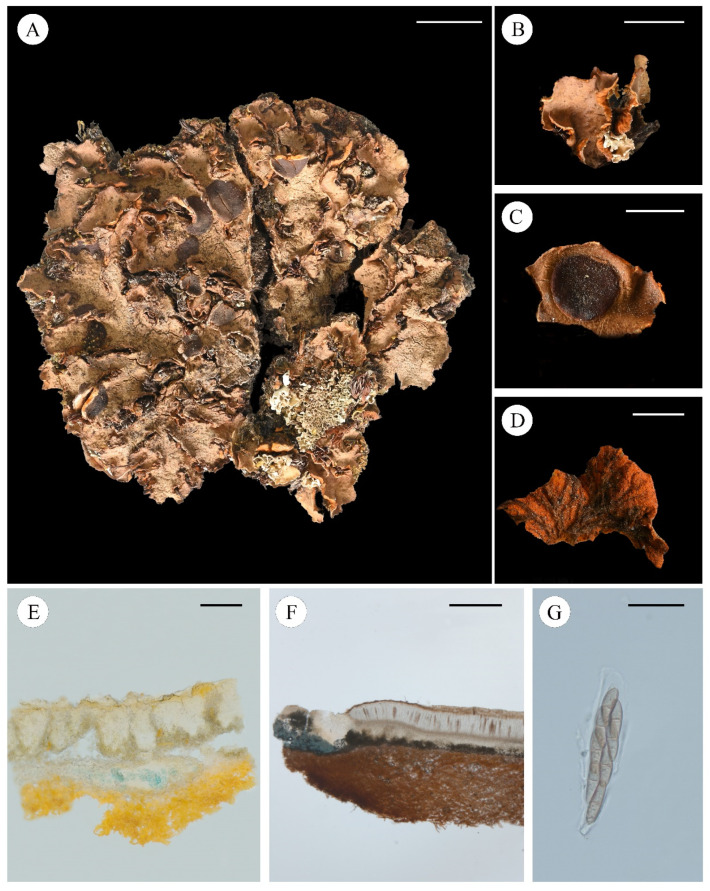
*Solorina crocea* ((**A**): Bruce McCune 18613 KUN, (**B**–**G**): Theodoer Esslinger 11831 KUN); (**A**), foliose thallus when dry; (**B**), smooth margin of the lobe; (**C**), upper surface of the apothecium; (**D**), bright orange lower surface distinctly brownish veined; (**E**), transversal sections of the thallus; (**F**), transversal sections of the apothecium; (**G**), ascus. Scale bars: 10 mm (**A**); 5 mm (**B**); 3 mm (**C**,**D**); 100 µm (**E**); 400 µm (**F**); 40 µm (**G**).

***Solorina crocea*** (L.) Ach., *K*. *Vetensk-Acad. Nya Handl.* 29: 228 (1808) **Figure 3**

≡ *Lichen croceus* L., *Sp. pl.* 2: 1149 (1753)

= *Peltigera crocea* (L.) Hoffm., *Descript. et Adumbr. Plant. Lich.* 2(3): 60 (1794)

= *Peltidea crocea* (L.) Ach., *Methodus*, Sectio post. (Stockholmiæ): 290 (1803)

= *Arthonia crocea* (L.) Ach., *Neues J. Bot.* 1(3. Stück): 20 (1806)

= *Parmelia crocea* (L.) Spreng., *Syst. veg.*, Edn 16 4(1): 280 (1827)

= *Solorina crocea* (L.) Ach., *Magy. Bot. Lapok*, 29: 30 (1930)

Type: LINN-HL1273-189 (lectotype, designated by Howe, *Bull. Torrey Bot. Club* 39: 201 (1912))

Description: Thallus: large foliose, wide-spreading, dorsiventral, heteromerous, lobate, up to 10 cm in diam.; Lobes: rounded, margins raised; Upper surface: olive green when wet, red-brown when dry, smooth to scabrid; Upper cortex: paraplectenchymatous, colorless or slightly yellow, measuring 70–150 µm; Medulla: 217–280 µm thick; Photobiont: comprises two photobiont layers, the upper layer contains *Coccomyxa*, exhibits a serrated shape and 40–110 µm thick, the lower layer, the *Nostoc* layer, is discrete, 20 or 70 µm thick; Lower cortex: lacking; Lower surface: bright orange, tomentose with a reticulate pattern of brown veins, with rhizoidal structures over 3 mm long, and internal cephalodia; Apothecia: large, rounded, irregularly scattered, not or rarely depressed in the upper surface, flat or convex, thalline margin absent; Asci: clavate, *Peltigera*-type, 8-spored; Ascospores: red-brown or dark brown, 1-septate with a median constriction, ellipsoid to fusiform, wall surface ornamented with rounded papillae, measuring 35–45 × 10–12 µm.

Chemistry: Solorinic acid, averantin, 6-*O*-methylaverantin and gyrophoric acid (detected by TLC and HPLC).

Ecology and distribution: Distributed in high-altitude areas and mainly grows on calcareous soil. Its widespread distribution includes: Alps [42], America [43], Canada [44], Finland [45], France [46], Germany [47], Greenland [48], Iberian Peninsula and Balearic Islands [49], Iceland [50], Ireland [51], Italy [52], Japan [53], New Zealand [54], Norway [55], Poland [56], Russia [57], Sweden [58], the Czech Republic [59], Ukraine [60].

Note: *S. crocea* is an easily recognizable species of *Solorina* due to the bright orange lower surface and the two layers of photobionts. This unique thallus structure serves as an identification characteristic. This species has a wide distribution and mainly occurs in the northern hemisphere.

Specimens examined: USA, Wyoming, Park Co., Alpine tundra, Beartooth Plateau North of West Summit of Beartooth Highway, Alt. 3150 m, July 1992, 44°59′ N, 109°26′ W, 1992, B. McCune, 18613; Whatcom Co.: vicinity of the Ross Lake overlook on N base of Ruby Mt.; ca. Alt. 2200 m in a Douglas Fir-lodgepole pine forest, in soil, 1980, Leg. Theodoer and L. Esslinger, 11831.

**Figure 4:**
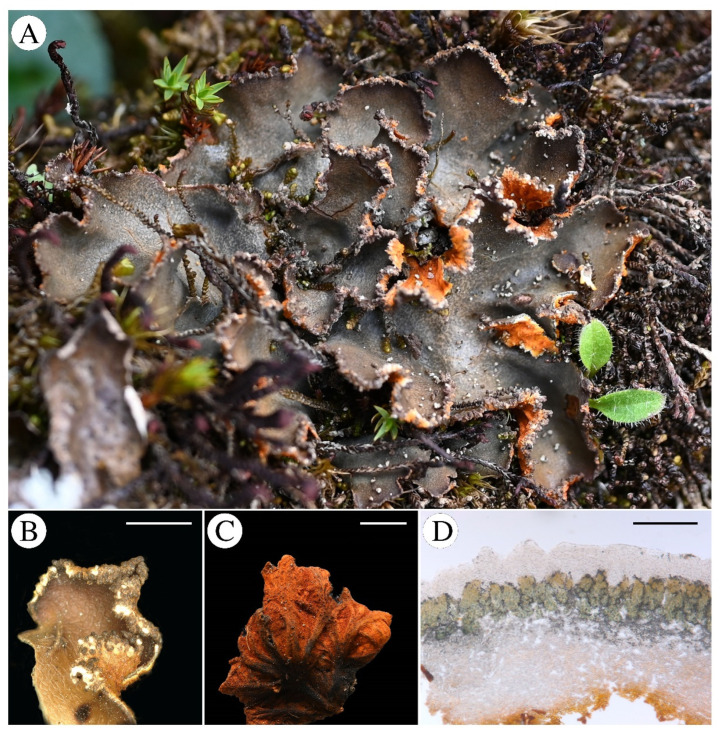
*Solorina crocoides* (T. Zheng 23-75450 KUN); (**A**), habit of *S. crocoides*; (**B**), margin of the lobe with black soredia; (**C**), bright orange lower surface distinctly brownish veined; (**D**), transversal sections of the thallus showing photobiont layer with both bright green chlorophyte cells and blue cyanobacteria cells. Scale bars: 2 mm (**B**); 5 mm (**C**); 200 µm (**D**).

***Solorina crocoides*** (Nyl.) Gyeln., *Magy. Bot. Lapok*, 29: 30 (1930) **Figure 4**

≡ *Solorinina crocoides* Nyl. *Naturaliste*, 6. Année: 387 (1884)

= *Solorina crocea* (L.) Ach. *Lichenology*, 4. 1–6 (2005)

Type: Himalaya, 3657 m (no date, no collector recorded), H-1662

Description: Thallus: large foliose, wide-spreading, dorsiventral, heteromerous, lobate, 2–8 cm in diam.; Lobes: rounded, margins raised, 5–15 mm wide, usually contiguous to adjacent or slightly overlapping; Upper surface: green-gray when wet, gray-brown when dry, smooth to scabrid, with soredia growing on the margin; Soredia: black, granulose; Upper cortex: paraplectenchymatous, 25–200 µm thick, colorless; Medulla: composed of variably orientated linear hyphae; Photobiont: a single photobiont layer c. 100–200 µm thick, mixed chlorophyte and cyanobacteria cells, contains *Asterochloris* and *Nostoc*; Lower cortex: lacks distinct lower cortex; Lower surface: bright orange, distinctly brownish veined, tomentose, and with clusters of simple or branched rhizines; Apothecia: not seen.

Chemistry: Solorinic acid, averantin, 6-*O*-methylaverantin (detected by TLC and HPLC).

Ecology and distribution: This species is typically found in high-altitude areas, primarily inhabiting calcareous soils on rocky substrates. It often coexists with herbaceous plants and bryophytes, with the absence of tall trees in the vicinity, resulting in favorable lighting conditions. It was reported from the Himalayan region [11] and China (Sichuan and Xizang).

Note: *Solorina crocoides* is similar to *S. crocea*. A distinguishing feature between these two species is that the upper surface of *S. crocea* appears olive green when wet, whereas *S. crocoides* is gray-brown. The thallus of *S. crocea* possesses smoother edges, and the thallus margin of *S. crocoides* has black soredia. Another difference lies in their photobiont layers—*S. crocea* has two separate photobiont layers, whereas *S. crocoides* features only one photobiont layer containing both chlorophytes and cyanobacteria.

Specimens examined: China, Xizang Prov., Linzhi city, Bomi Co., 29°46′16.93″ N, 95°41.’57.39″ E, Alt. 3903 m, 9 August 2023, T. Zheng, 23-75455, 23-75456, 23-75432, 23-75431, 23-75453, 23-75450; 29°45′57.53″ N, 95°41′50.64″ E, Alt. 3798 m, 9 August 2023, T. Zheng, 23-75433, 23-75434, 23-75437; 29°46′16.93″ N, 95°41′57.39″ E, Alt. 3839 m, 9 August 2023, T. Zheng, 23-75449; 29°45.630′ N, 95°42.052′ E, Alt. 4065 m, 19 September 2014, L. S. Wang et al., 14-45996. Sichuan Prov., Luding Co. Gongga Mt., 29°20′ N, 101°30′ E, Alt. 3000 m, on rock, 1996, L. S. Wang, 96-16177.

***Pseudosolorina*** T. Zheng and Li S. Wang, gen. nov.

Fungal Names: FN 572274

Type species: *Pseudosolorina parmigera* T. Zheng and Li S. Wang, sp. nov.

Etymology: Referring to its similarity to *Solorina*.

Diagnosis: This genus has scattered round, immersed, slightly to very deeply concaved apothecia without a thalline margin. Thallus foliose, lower surface white or pale brown, indistinctly veined, it is similar to *Solorina* in external morphology, but differs by the white to pale brown lower surface.

Description: Thallus: small to large foliose, dorsiventral, heteromerous, lobate; Lobes: rounded, margins flush with the substrate or slightly raised; Upper surface: apple green to green-gray when wet, white or pale-green to brown when dry, smooth to scabrid; Upper cortex: paraplectenchymatous; Photobiont: *Coccomyxa* and *Nostoc*; Lower cortex: lacking; Lower surface: white or pale brown, indistinctly veined, tomentose, and with clusters of simple or branched rhizines; Apothecia: rounded, irregularly scattered, impressed to immersed in the upper surface, red-brown to black, slightly to very deeply concave, thalline margin absent; Asci: clavate, *Peltigera*-type, 1-, 2- or 4-spored; Ascospores: red-brown or dark brown, 1- or 2-(3-)septate with median constriction, ellipsoid to fusiform, wall non-uniformly thickened, surface ornamented broad reticulating ridges and irregular lacunae.

Chemistry: Two species contain gyrophoric acid, methyl gyrophorate, tenuiorin, and 2′-*O*-methyltenuiorin, but six species lack secondary metabolites (detected by TLC and HPLC).

Ecology: On soil, markedly calcicole. The members of this genus often occur in alpine habitats.

Note: Previously, species of this genus were classified under *Solorina* based on the morphology of the apothecia. However, phylogenetic analyses indicate strong support for its status as a monophyletic genus. *Pseudosolorina* is set apart from related genera by its white or pale brownish lower surface, with apothecia irregularly scattered and immersed in the upper surface of the thallus, usually concave. In comparison to *Solorina*, its spore wall is rougher and ornamented with broad reticulating ridges and irregular lacunae. Most species lack secondary metabolites.

**Figure 5:**
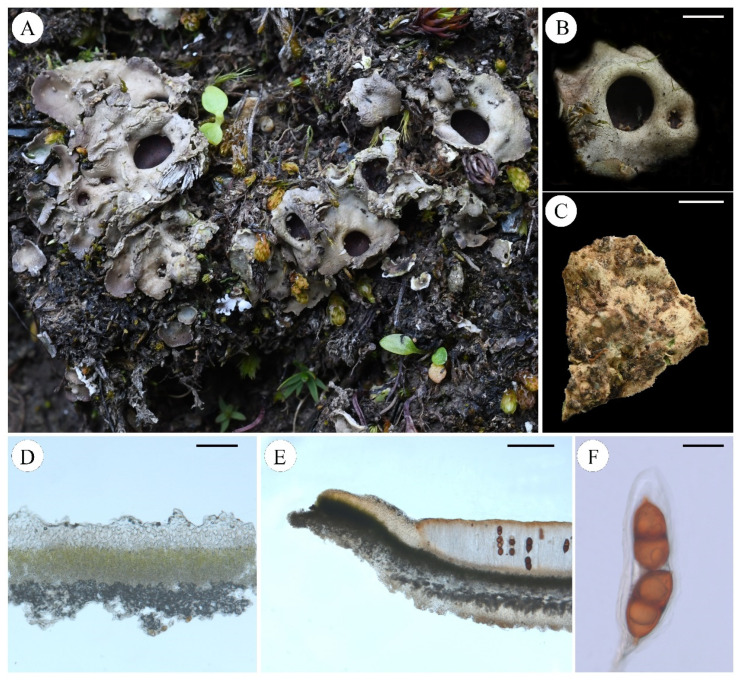
*Pseudosolorina bispora* ((**A**): L. S. Wang et al. 20-66618 KUN, (**B**): M. Ai 24-76716 KUN, (**C**–**F**): Y. Y. Zhang ZYY-46 KUN); (**A**), habit of *P*. *bispora*; (**B**), apothecium; (**C**), white lower surface with brownish tomentum; (**D**), transversal sections of the thallus; (**E**), transversal sections of the apothecium; (**F**), ascus. Scale bars: 2 mm (**B**,**C**); 100 µm (**D**); 300 µm (**E**); 40 µm (**F**).

***Pseudosolorina bispora*** (Nyl.) T. Zheng and Li S. Wang, comb. nov. **Figure 5**

≡ *Solorina bispora* Nyl., *Syn. meth. lich.* (Parisiis) 1(2): 331 (1860)

= *Solorina saccata* var. bispora (Nyl.) Arnold, *Verh. Kaiserl.-Königl. Zool.-Bot. Ges. Wien*, 21(3–4): 1118 (1871)

= *Solorina bispora* Nyl., *Magy. Bot. Lapok*, 29: 29 (1930)

Fungal Names: FN 572279

Type: France, Midi-Pyrénées, Hautes-Pyrénées, Nylander W., H9506085 (Holotype)

Description: Thallus: small foliose, fragile, dorsiventral, heteromerous, lobate, 1.5–2 cm in diam.; Lobes: rounded or irregular, 5–10 mm; Upper surface: bright apple-green when wet, dark green or pale grey to tinged brown when dry, usually covered with white-pruinose; Upper cortex: paraplectenchymatous, colorless, 50–100 μm thick; Medulla: 40–150 μm thick; Photobiont: *Coccomyxa* and *Nostoc*, one *Coccomyxa* green algal layer, 40–110 μm thick, *Nostoc* present in internal cephalodia which can be seen on the underside of the thallus as dark spots; Lower cortex: lacking; Lower surface: pale brown, densely tomentose, not or indistinctly veined, rhizines; Apothecia: large, rounded, irregularly scattered, sunk in depressions in the upper surface, red-brown to black, deeply concave, thalline margin absent; Asci: clavate, *Peltigera*-type, 2-spored; Ascospores: red-brown or dark brown, 1-septate with a median constriction, ellipsoid to fusiform, wall non-uniformly thickened, surface ornamented broad reticulating ridges and irregular lacunae, 65–88 × 33–42 µm.

Chemistry: No lichen products detected by TLC.

Ecology and distribution: Typically found in humid soil rich in humus, often growing in association with herbaceous plants. Distributed in Alps [42], America [61], Canada [44], France [6,46], Germany [47], Greenland [62], Iberian Peninsula and Balearic Islands [49], Italy [52], New Mexico [63], Norway [64], Poland [56], Ukraine [60], and China.

Note: This species resembles *Pseudosolorina saccata* and was previously classified as a high-altitude variant of *P. saccata*. In comparison to *P. saccata*, its thallus is relatively small (<2 cm in diam.), often exhibiting a covering of pruina; it has two ascospores.

Specimens examined: Austria, Salzburg, Tamsweg, Grossek-Speiereck, 47°7′54″ N, 13°38′17″ E, Alt. 2162 m, 31 August 2019, Y. Y. Zhang, ZYY-46; China, Sichuan Prov., Hongyuan city, 32°13′45.83″ N, 102°35′44.75″ E, Alt. 4278 m, 9 April 2020, L. S. Wang et al., 20-66618; Yunnan Prov., Kunming city, Tangdan town, Yaojingtang, 26°15′27″ N, 102°95′79″ E, Alt. 4000 m, 15 September 2024, M. Ai, 24-76716.

**Figure 6:**
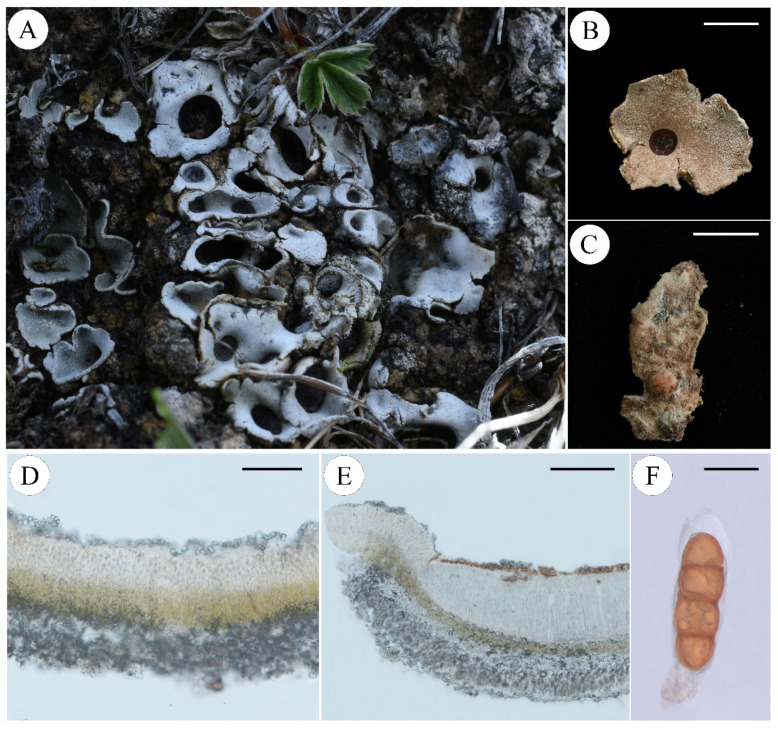
*Pseudosolorina bispora* var. *monospora* (L. S. Wang et al. 20-67082 KUN); (**A**), habit of *P*. *bispora* var. *monospora*; (**B**), apothecium; (**C**), white lower surface with brownish tomentum; (**D**), transversal sections of the thallus; (**E**), transversal sections of the apothecium; (**F**), ascus. Scale bars: 2 mm (**B**); 3 mm (**C**); 100 µm (**D**); 200 µm (**E**); 40 µm (**F**).

***Pseudosolorina bispora* var.** ***monospora*** (Gyeln.) T. Zheng and Li S. Wang, comb. nov. **Figure 6**

≡ *Solorina monospora* Gyeln., *Magy. Bot. Lapok*, 29: 29 (1930)

= *Solorina bispora* var. *monospora* (Gyeln.) Frey, *Ergebn. wiss. Unters. schweiz. NatnParks*, N.S. 3(27): 377 (1952).

Fungal Names: FN 572281

Description: Thallus: small foliose, fragile, dorsiventral, heteromerous, lobate, *ca* 1 cm in diam.; Lobes: rounded or irregular, 2–8 mm; Upper surface: bright apple-green when wet, dark green or pale grey to tinged brown when dry, usually covered with white pruina, pale grey to brown-grey, often pruinose; Upper cortex: paraplectenchymatous, colorless, 40–80 μm thick; Medulla: 40–150 μm thick; Photobiont: *Coccomyxa* and *Nostoc*, one *Coccomyxa* green-algal layer, c. 20–80 μm thick, *Nostoc* exist in internal cephalodia which can be seen on the underside of the thallus as dark spots; Lower cortex: lacking; Lower surface: pale brown, densely tomentose, not or indistinctly veined, rhizines, cephalodia internal, rarely external; Apothecia: large, rounded, irregularly scattered, sunk in depressions in the upper surface, red-brown to black, deeply concave, thalline margin absent; Asci: clavate, *Peltigera*-type, 1-spored; Ascospores: red-brown or dark brown, 2- (3-) septate with median constriction, ellipsoid to fusiform, wall non-uniformly thickened, surface ornamented with broad reticulating ridges and irregular lacunae, 100–160 × 35–50 µm.

Chemistry: No lichen products detected by TLC.

Ecology and distribution: This species is typically found in alpine environments, where it grows on basic soils formed over shaded limestone or calcareous schists. It is distributed in France [46], Iberian Peninsula and Balearic Islands [49], Switzerland [65], and China.

Note: This species closely resembles a variant of *P. bispora* (= *Solorina bispora* var. *monospora* (Gyeln.) Frey) in appearance, containing only 1 spore in the ascus. However, phylogenetic analysis suggests that it should be recognized as an independent species. Unfortunately, we did not obtain the type specimen or molecular data related to the origin of *S. bispora* var. *monospora*, so we can only describe this specimen as a similar species to *S. bispora* var. *monospora*, revised to *Pseudosolorina bispora* var. *monospora* (Gyeln.) T. Zheng and Li S. Wang. Further research is necessary to determine whether they are indeed the same species.

Specimens examined: China, Qinghai Prov., Zhiduo Co., 33°48′03.32″ N, 95°13′57.24″ E, Alt. 4436 m, 18 September 2020, X. Y. Wang et al., XY20-828; Maduo Co., 35°06′16.62″ N, 98°54′22.97″ E, Alt. 4260 m, 14 September 2020, X. Y. Wang et al., XY20-2943; Maduo Co., 34°49′40.56″ N, 99°02′03.48″ E, Alt. 4578 m, 13 September 2020, L. S. Wang et al., 20-67082.

**Figure 7:**
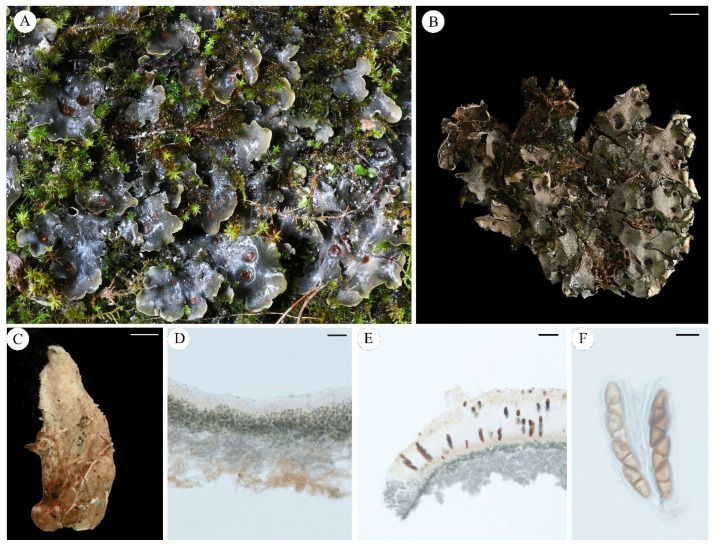
*Pseudosolorina hepatizon* (L. S. Wang et al. 23-75286 KUN); (**A**), habit of *P*. *hepatizon*; (**B**), foliose thallus when dry; (**C**), white lower surface with brownish tomentum and rhizines; (**D**), transversal sections of the thallus; (**E**), transversal sections of the apothecium; (**F**), ascus. Scale bars: 10 mm (**B**); 2 mm (**C**); 50 µm (**D**); 100 µm (**E**); 20 µm (**F**).

***Pseudosolorina hepatizon*** T. Zheng and Li S. Wang, sp. nov. **Figure 7**

Fungal Names: FN 572275

Type: China. Yunnan Prov., Shangrila city, Birong Valley, 26°39′05.72″ N, 99°44′15.57″ E, Alt. 3089 m, 15 July 2023, L. S. Wang et al., 23-75286 (Holotype KUN)

Etymology: Referring to its hepaticolor thallus.

Diagnosis: This species has a hepaticolor upper surface when dry, while most other species of *Pseudosolorina* display brown, greyish-brown, or pale hues. *P. hepatizon* has a single photobiont layer containing both cyanobacteria and chlorophytes and lacks secondary metabolites.

Description: Thallus: large foliose, dorsiventral, heteromerous, lobate, up to 9 cm in diam.; Lobes: rounded, 10–20 mm; Upper surface: green-gray when wet, hepaticolor or gray-brown when dry, smooth to scabrid; Upper cortex: paraplectenchymatous, colorless, 60–150 μm thick; Medulla: 200–300 μm thick; Photobiont: *Asterochloris* and *Nostoc*, single photobiont layer with both blue and bright green cells, 100–220 μm thick; Lower cortex: lacking; Lower surface: white, indistinctly veined, brownish tomentose, without cephalodia, with clusters of simple or branched rhizines; Apothecia: large, rounded, irregularly scattered, sunk in depressions in the upper surface, red-brown to black, slightly concave, thalline margin absent; Asci: clavate, *Peltigera*-type, 4-spored; Ascospores: red-brown or dark brown, 1-septate with a median constriction, ellipsoid to fusiform, wall non-uniformly thickened, surface ornamented broad reticulating ridges and irregular lacunae, measured 42–53 × 18–25 µm.

Chemistry: No lichen products detected by TLC.

Ecology and distribution: It is distributed in high-altitude areas and primarily found on calcareous soils on stones, typically growing alongside or on bryophytes. This species is only known from Yunnan Province of China.

Note: Specimens representing this species were formerly categorized as *Solorina simensis* based on their morphological characteristics. In this study they have been divided from *P. simensis* to form the new species *P. hepatizon*. Despite distinct chemical characteristics, *P. simensis* contains secondary metabolites such as methyl gyrophorate and tenuiorin, whereas *P. hepatizon* lacks any secondary metabolites; it was previously identified as an acid-deficient variant of *S. simensis* (Krog and Swinscow 1986). DNA evidence supports its classification as a distinct species in the realm of scientific research.

Specimens examined: China, Yunnan Prov., Kunming city, Dongchuan Dist., Guniuzhai Mt., 26°09′50.25″ N, 103°13′30.86″ E, Alt. 3100 m, 10 May 2017, L. S. Wang et al., 17-55094; Shangrila city, Birong Valley, 26°39′05.72″ N, 99°44′15.57″ E, Alt. 3089 m, 15 July 2023, L. S. Wang et al., 23-75286.

**Figure 8:**
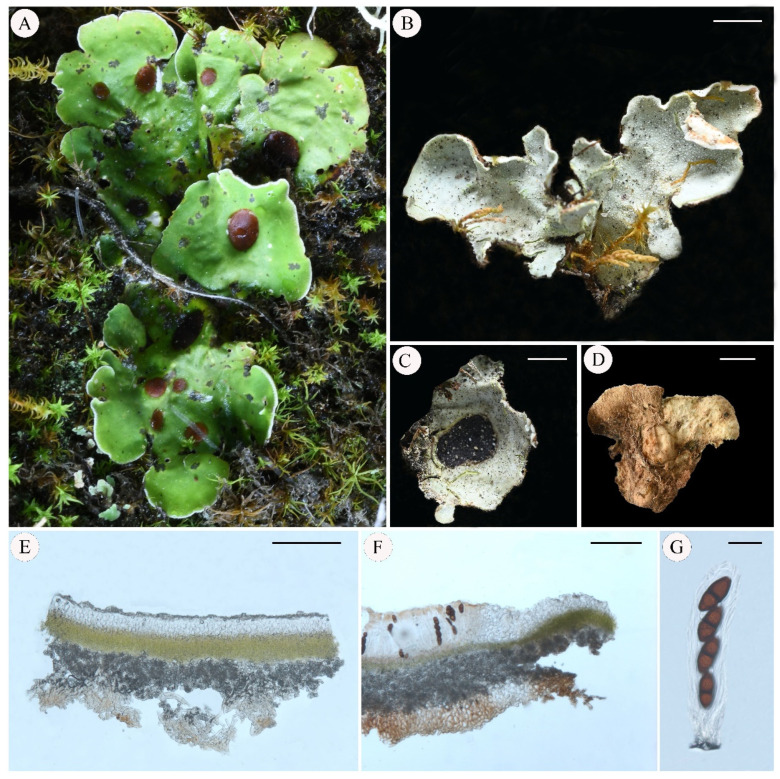
*Pseudosolorina parmigera* (**A**: L. S. Wang et al. 23-75751 KUN, **B**,**C**: L. S. Wang and M. Ai 24-76096 KUN, (**D**–**G**) X. Y. Wang et al. 21-70423 KUN); (**A**), habit of *P*. *parmigera*; (**B**), upper surface with white pruina; (**C**), apothecium; (**D**), white lower surface with brownish tomentum and rhizines; (**E**), transversal sections of the thallus; (**F**), transversal sections of the apothecium; (**G**), ascus. Scale bars: 2 mm (**B**,**D**); 3 mm (**C**); 200 µm (**E**,**F**); 40 µm (**G**).

***Pseudosolorina parmigera*** T. Zheng and Li S. Wang, sp. nov. **Figure 8**

Fungal Names: FN 572277

Type: China, Yunnan Prov., Shangrila city, road side of x236, 26°38′01.25″ N, 99°43′07.92″ E, Alt. 3035 m, 15 July 2023, L. S. Wang et al., 23-75751 (Holotype KUN)

Etymology: Referring to its shield-like shaped thallus.

Diagnosis: This species has a shield-like shape of the thallus and one green photobiont layer similar to *P. saccata*, but *P. parmigera* has extensive white pruina and no external cephalodia on the upper surface, whereas *P. saccata* has external cephalodia and rarely white pruina on the upper surface.

Description: Thallus: large foliose, fragile, dorsiventral, heteromerous, lobate, 2–6 cm in diam.; Lobes: rounded, 5–15 mm; Upper surface: bright apple-green when wet, dark green to pale grey to tinged brown when dry, white-pruinose, smooth to scabrid; Upper cortex: paraplectenchymatous, colorless, 30–80 μm thick; Medulla: 100–250 μm thick; Photobiont: *Coccomyxa* and *Nostoc*, single *Coccomyxa* green-algal layer, c. 40–110 μm thick, *Nostoc* present in internal cephalodia which can be seen on the underside of the thallus as dark spots; Lower cortex: lacking; Lower surface: pale brown, densely tomentose, not or indistinctly veined, rhizines abundant, length: 10 mm; Apothecia: large, rounded, irregularly scattered, sunk in depressions in the upper surface, red-brown to black, medially concave, thalline margin absent; Asci: clavate, *Peltigera*-type, 4-spored; Ascospores: red-brown or dark brown, 1-septate with a median constriction, ellipsoid to fusiform, wall non-uniformly thickened, surface ornamented broad reticulating ridges and irregular lacunae, dimensions: 35–50 × 17–23 µm.

Chemistry: No lichen products detected by TLC.

Ecology and distribution: Growing in calciferous soil rich in humus and terricolous mosses at approximately 3000 m in the alpine zone. This species is known from Yunnan, Sichuan, and Qinghai Provinces of China.

Note: This species shares the most similar morphological and chemical characteristics with *P. saccata*; the difference between these two species is that *P. saccata* has external cephalodia and is rarely pruinose on the upper surface, but the thallus of *P. parmigera* has dense pruina and lacks external cephalodia on the upper surface.

Specimens examined: China, Yunnan Prov., Lijiang city, Yulong Snow Mt. 27°07′36.82″ N, 100°13′40.70″ E, Alt. 2938 m, 8 July 2019, L. S. Wang et al., 19-62636; Shangrila city, road side of x224, 26°38′07.56″ N, 99°43′00.12″ E, Alt. 3062 m, 15 July 2023, L. S. Wang et al., 23-75739, 23-75734; Shangrila city, road side of x236, 26°38′01.25″ N, 99°43′07.92″ E, Alt. 3035 m, 15 July 2023, L. S. Wang et al., 23-75751; Dali city, Cangshan National Geopark, 25°41′42.47″ N, 100°06′30.73″ E, Alt. 3075 m, 19 May 2024, L. S. Wang and M. Ai, 24-76096. Sichuan Prov., Liangshan Yi Autonomous Prefecture Muli Co. 27°41′51.41″ N, 101°13′52.86″ E, Alt. 3177 m, 10 September 2021, X. Y. Wang et al., 21-70363, 21-70423, XY21-272, XY21-275. Qinghai Prov., Menyuan Co., 37°12′34″ N, 102°0′39″ E, Alt. 2711 m, 24 July 2022, S. B. Zhang et al., ZSB22-133; Manma Co., 32°46′50″ N, 101°11′2″ E, Alt. 3553 m, 14 July 2022, S. B. Zhang et al., ZSB22-256.

**Figure 9:**
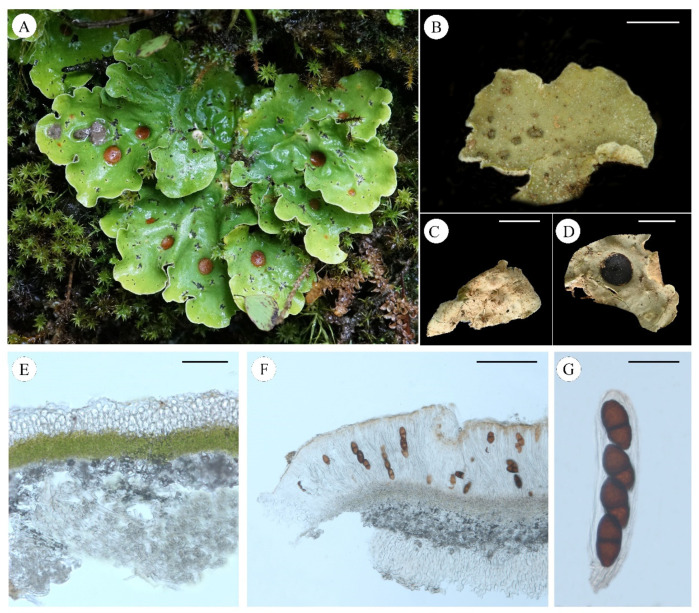
*Pseudosolorina saccata* ((**A**): X. Y. Wang and YX Gan XY23-383 KUN, (**B**): L. S. Wang et al. 23-75281 KUN, **C**–**G**: L. S. Wang et al. 23-75757 KUN); (**A**), habit of *P*. *saccata*; (**B**), upper surface with external cephalodia; (**C**), white lower surface with rhizines; (**D**), apothecium; (**E**), transversal sections of the thallus; (**F**), transversal sections of the apothecium; (**G**), ascus. Scale bars: 1 mm (**B**); 4 mm (**C**); 3 mm (**D**); 100 µm (**E**); 200 µm (**F**); 40 µm (**G**).

***Pseudosolorina saccata*** (L.) T. Zheng and Li S. Wang, comb. nov. **Figure 9**

≡ *Lichen saccatus* L., *Fl. Suec.*, Edn 2: 419 (1755)

= *Lobaria saccata* (L.) Hoffm., *Deutschl. Fl.*, Zweiter Theil (Erlangen): 147 (1796)

= *Peltidea saccata* (L.) Ach., *Methodus*, Sectio post. (Stockholmiæ): 290 (1803)

= *Peltigera saccata* (L.) DC., in Lamarck and de Candolle, *Fl. franç.*, Edn 3 (Paris) 2: 408 (1805)

= *Arthonia saccata* (L.) Ach., *Neues J. Bot.* 1(3. Stück): 21 (1806)

= *Solorina saccata* (L.) Ach., *K. Vetensk-Acad. Nya Handl.* 29: 228 (1808)

= *Platysma saccatum* (L.) Frege, *Deutsch. Botan. Taschenb.* 2: 165 (1812)

= *Solorina saccata* (L.) Ach., *Magy. Bot. Lapok*, 29: 29 (1930)

Fungal Names: FN 572280

Type: LINN-HL1273-197 (Lectotype, designated by Jørgensen et al., *Bot. J. Linn. Soc.* 115: 381. 1994)

Description: Thallus: large foliose, fragile, dorsiventral, heteromerous, lobate, 3–6 cm in diam.; Lobes: rounded, 4–20 mm; Upper surface: bright apple-green when wet, dark green to pale grey to tinged brown when dry, white-pruinose, having external cephalodia; Upper cortex: paraplectenchymatous, colorless, 50–100 μm thick; Medulla: 200–350 μm thick; Photobiont: *Coccomyxa* and *Nostoc*, single *Coccomyxa* green-algal layer, c. 50–110 μm thick, *Nostoc* present in external cephalodia on the upper surface and internal cephalodia which can be seen on the underside of the thallus as dark spots; Lower cortex: lacking; Lower surface: pale brown, densely tomentose, not or indistinctly veined, rhizines abundant and long, up to 18 mm; Apothecia: large, rounded, irregularly scattered, sunk in depressions in the upper surface, red-brown to black, medially concave, thalline margin absent; Asci: clavate, *Peltigera*-type, 4-spored; Ascospores: red-brown or dark brown, 1-septate with a median constriction, ellipsoid to fusiform, wall non-uniformly thickened, surface ornamented with broad reticulating ridges and irregular lacunae, dimensions: 32–60 × 18–27 µm.

Chemistry: No lichen products detected by TLC.

Ecology and distribution: This species grows in calcareous soil rich in humus and terrestrial mosses in a cool-temperate alpine region, often inhabiting rock crevices and also commonly found along the edges of groves. Its widespread distribution includes: Alps [52], USA [66], Canada [44], England [67], France [46], Germany [47], Greece [68], Iberian Peninsula and Balearic Islands [49], India [69], Italy [52], Japan [53], Norway [64], Poland [56], Siberia [70], the Czech Republic [59], Ukraine [60] and China.

Note: *Pseudosolorina saccata* is widely distributed and morphologically variable. *P. simensis* and *P. bispora* were previously classified as variants under this species. *P. saccata* is characterized by a green upper surface, a white lower surface, a concave apothecium, and 4-spored ascus. Notably, it closely resembles *P. parmigera*; however, distinct attributes for *P. saccata* include external cephalodia and rare white pruina on the upper surface of the thallus, whereas *P. parmigera* typically exhibits a white pruinose covering on its upper surface and lacks external cephalodia.

Specimens examined: China, Yunnan Prov., Shangrila city, Birong Valley, 26°39′04.17″ N, 99°44′13.48″ E, Alt. 3082 m, 15 July 2023, L. S. Wang et al., 23-75282; road side of X241, 26°37′54.86″ N, 99°43′14.07″ E, Alt. 3051 m, 15 July 2023, L. S. Wang et al., 23-75756, 23-75757, 23-75755; Birong Valley, 26°39′03.98″ N, 99°44′13.12″ E, Alt. 3486 m, 15 July 2023, X. Y. Wang and Y. X. Gan, XY23-383; Birong Valley, 26°39′04.26″ N, 99°44′13.77″ E, Alt. 3047 m, 15 July 2023, L. S. Wang et al., 23-75281(a), 23-75280; Dali city, Cangshan National Geopark, 25°41′41.09″ N, 100°06′32.38″ E, Alt. 3070 m, 19 May 2024, L. S. Wang et al., 24-76098 (b). Qinghai Prov., Xunhua Co., 35°41′48″ N, 102°26′26.18″ E, Alt. 2688 m, 6 August 2022, S. B. Zhang and H. L. Kang, ZSB22-67; Banma Co., 32°47′15″ N, 101°4′45″ E, Alt. 3680 m, 14 July 2022, S. B. Zhang and H. L. Kang, ZSB22-263; Huzhu Co., 36°44′41″ N, 102°32′47″ E, Alt. 2759 m, 16 August 2022, S. B. Zhang and H. L. Kang, ZSB22-340. Gansu Prov., Luqu Co., 34°08′27.58″ N, 102°11′57.16″ E, Alt. 3588 m, 9 July 2022, L. S. Wang et al., 22-73302, 22-73303. Sichuan Prov., Jiulong Co., Wuxuhai Lake, 29°09′06.14″ N, 101°24′42.58″ E, Alt. 3691 m, 9 June 2023, L. S. Wang et al., 23-75120.

**Figure 10:**
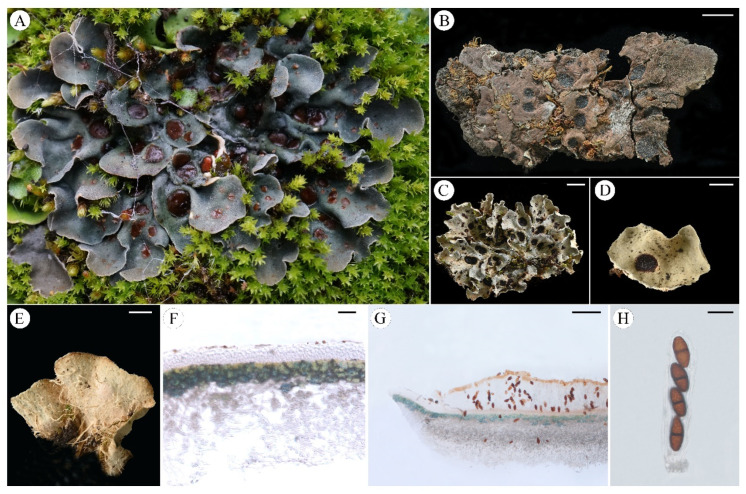
*Pseudosolorina simensis* (**A**,**C**: X. Y. Wang et al. XY21-274 KUN, **B**: W. Schimper 32,914 H-NYL, (**D**–**G**): X. Y. Wang et al. 21-70422 KUN); (**A**), habit of *P*. *simensis*; (**B**), type of *P. simensis*; (**C**), foliose thallus when dry; (**D**), apothecium; (**E**), white lower surface with rhizines; (**F**), transversal sections of the thallus; (**G**), transversal sections of the apothecium; (**H**), ascus. Scale bars: 5 mm (**B**,**C**); 2 mm (**D**,**E**); 50 µm (**F**); 200 µm (**G**); 30 µm (**H**).

***Pseudosolorina simensis*** (Hochst. ex Flot.) T. Zheng and Li S. Wang, comb. nov. **Figure 10**

≡ *Solorina simensis* Hochst. ex Flot., *Linnaea* 17: 17 (1843)

= *Solorina saccata* var. *simensis* (Hochst. ex Flot.) Nyl., *Mém. Soc. Imp. Scity Nat. Cherbourg*, 5: 101 (1858)

= *Solorinina simensis* (Hochst. ex Flot.) Nyl., *Naturaliste*, 6. Année: 387 (1884)

= *Solorina simensis* Hochst. ex Flot., *Magy. Bot. Lapok*, 29: 29 (1930)

Fungal Names: FN 572282

Type: Ethiopia, Amhara Region, Simien Mountains, W. Schimper, H-NYL 32,912 (Holotype H-NYL)

Description: Thallus: large foliose, fragile, flat, dorsiventral, heteromerous, lobate, *ca* 5 cm in diam.; Lobes: rounded, 5–8 mm; Upper surface: light blue when wet, gloomy to brown when dry, rarely pruinose on the margin; Upper cortex: paraplectenchymatous, colorless, 40–80 μm thick; Medulla: pale, 200–280 μm thick; Photobiont: *Asterochloris* and *Nostoc*, both present in a single photobiont layer, 70–150 μm thick in thallus and 60–100 µm in apothecium; Lower cortex: lacking; Lower surface: pale to brown, indistinctly veined, tomentose, without cephalodia, with clusters of simple or branched rhizines; Apothecia: large, rounded, irregularly scattered, sunk in depressions in the upper surface, red-brown to black, slightly concave, thalline margin absent; Asci: clavate, *Peltigera*-type, 4-spored; Ascospores: red-brown or dark brown, 1-septate with a median constriction, ellipsoid to fusiform, wall non-uniformly thickened, surface ornamented with broad reticulating ridges and irregular lacunae, dimensions: 35–65 × 16–23 µm.

Chemistry: Gyrophoric acid, methyl gyrophorate, tenuiorin, and 2′-*O*-methyltenuiorin (detected by TLC and HPLC).

Ecology and distribution: It grows in high-altitude areas and is mainly found in calcareous soil on stone, usually growing together with or on bryophytes. It is distributed in East Africa [71], Ethiopia [5], Mexico [72], New Guinea [73], and China.

Note: This species could be distinguished by its morphological characters of the thallus and the single photobiont layer with both cyanobacterial and chlorophyte cells. It is similar to *P. tenuior*, but *P. simensis* has a thicker photobiont layer up to 150 μm in thallus and 100 µm in apothecium.

Specimens examined: Ethiopia, Amhara Region, Simien Mountains, W. Schimper, H-NYL 32,912 (holotype); H-NYL 32914. China, Sichuan Prov., Liangshan Yi Autonomous Prefecture, Muli Co., 27°43′30.82″ N, 101°14′11.52″ E, Alt. 3010 m, 11 September 2021, X. Y. Wang et al., XY21-274, 21-70422; Litang Co., 30°25′30.27″ N, 101°18′07.81″ E, Alt. 3296 m, 12 July 2022, D. Liu, LD22-696, LD22-697; Yunnan Prov., Shangrila city, road side of X246, 26°37′56.13″ N, 99°43′31.28″ E, Alt. 3110 m, 15 July 2023, L. S. Wang et al., 23-75761.

**Figure 11:**
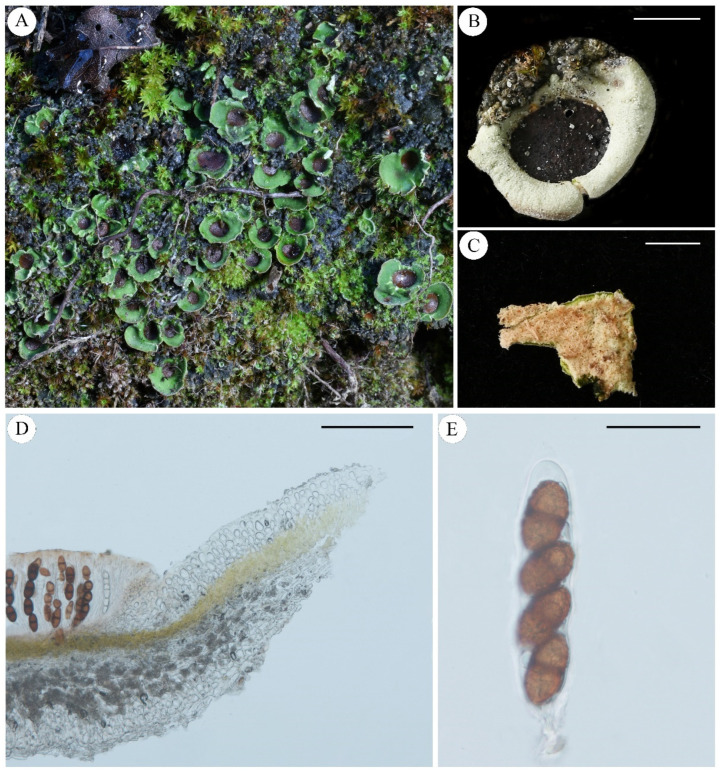
*Pseudosolorina spongiosa*, (**A**: L. S. Wang et al. 24-76099 KUN, **B**–**E**: L. S. Wang et al. 23-75119 KUN); (**A**), habit of *P*. *spongiosa*; (**B**), apothecium; (**C**), white lower surface with brownish tomentum; (**D**), transversal sections of the apothecium; (**E**), ascus. Scale bars: 2 mm (**B**); 1 mm (**C**); 200 µm (**D**); 40 µm (**E**).

Note: This species exhibits morphological similarities with *Solorina embelina*, particularly in their thallus structure, where reduced thalli form a distinct small circle around the apothecium. However, the two species differ in terms of spore number: *S. embelina* contains only one spore per ascus, whereas *P. spongiosa* possesses four spores per ascus. Through a meticulous review and comparison of the original literature on *Lichen spongiosus*, we have identified discrepancies between the initial documentation of this species and the present specimens. The original records describe the species as having a clustered and branched thallus; apothecia that are scattered, concave, brown, externally spongy, and pale, with a thin, upright margin. In contrast, current samples of *P. spongiosa* feature a small thallus, lacking lobes and apothecia that are scattered, concave, brown to black, without any margin. However, due to limited access to the holotype of *Lichen spongiosus*, we are unable to define this species. However, our samples are phylogenetically and morphologically identical to the currently accepted species *Solorina spongiosa*. Consequently, we have opted to retain the classification of this species as *P. spongiosa*.

***Pseudosolorina spongiosa*** (Sm.) T. Zheng and Li S. Wang, comb. nov. **Figure 11**

≡ *Lichen spongiosus* Sm., in Smith and Sowerby, *Engl. Bot.* 20: tab. 1374 (1805)

= *Collema spongiosum* Ach., *Lich. Univ.*: 661 (1810)

= *Lichen furvus* * *spongiosum* (Ach.) Lam., *Encycl. Méth. Bot.*, Suppl. (Paris) 3(2): 416 (1813)

= *Polychidium spongiosum* (Ach.) Gray, *Nat. Arr. Brit. Pl.* (London) 1: 402 (1821)

= *Lecanora limbata* Sommerf., *Suppl. Fl. lapp.* (Oslo): 105 (1826)

= *Parmelia spongiosa* (Ach.) Spreng., *Syst. veg.*, Edn 16 4(1): 277 (1827)

= *Peltigera saccata* var. *limbata* (Sommerf.) Fr., *Summa veg. Scand.*, Sectio Prior (Stockholm): 104 (1845)

= *Solorina saccata* ß *limbata* (Sommerf.) Schaer., *Enum. critic. lich. europ.* (Bern): 23 (1850)

= *Peltigera limbata* (Sommerf.) Nyl., *Not. Sällsk. Fauna et Fl. Fenn. Förh.* 2: 217 (1852)

= *Solorina saccata* f. *limbata* (Sommerf.) Nyl., *Mém. Soc. Imp. Sci. Nat. Cherbourg* 3: 173 (1855)

= *Solorina saccata* var. *spongiosa* (Ach.) Nyl., *Syn. meth. lich.* (Parisiis) 1(2): 331 (1860)

= *Solorina limbata* (Sommerf.) Mudd, *Man. Brit. Lich.*: 85 (1861)

= *Solorina spongiosa* (Sm.) Anzi, *Comm. Soc. crittog. Ital.* 1(fasc. 3): 136 (1862)

= *Solorinina simensis* var. *limbata* (Sommerf.) Nyl., *in Hue*, *Nouv. Arch. Mus. Hist. Nat.*, Paris, 3 sér. 2: 312 (1890)

= *Solorina spongiosa* (Sm.) Anzi, *Magy. Bot. Lapok*, 29: 29 (1930)

Fungal Names: FN 572276

Type: BM000975901 (type, collected by Anon. on 27 April 1803)

Diagnosis: This species has a reduced thallus, often with white pruina; this morphological character is shared with *Solorina embelina*, but *P. spongiosa* has four spores per ascus, whereas *Solorina embelina* only has one.

Description: Thallus: foliose, fragile, dorsiventral, heteromerous, poorly developed, restricted to a continuous or lacerate narrow rim surrounding the apothecium, 0.1–0.7 cm in diam.; Upper surface: bright green when wet, dark green to pale grey when dry, usually covered with white pruina; Upper cortex: paraplectenchymatous, colorless, 10–20 μm thick; Medulla: 40–80 μm thick; Photobiont: *Coccomyxa* and *Nostoc*, one *Coccomyxa* green-algal layer, 20–60 μm thick, *Nostoc* present in internal cephalodia which can be seen on the underside of the thallus as dark spots; Lower cortex: lacking; Lower surface: pale brown, densely tomentose, not or indistinctly veined, rhizines; Apothecia: rounded, irregularly scattered, sunk in depressions in the upper surface, red-brown to black, medially concave, thalline margin absent; Asci: clavate, *Peltigera*-type, 4-spored; Ascospores: red-brown or dark brown, 1-septate with a median constriction, ellipsoid to fusiform, wall non-uniformly thickened, surface ornamented broad reticulating ridges and irregular lacunae, measured 30–50 × 18–23 µm.

Chemistry: No lichen products detected by TLC.

Ecology and distribution: This species typically thrives in moist calcareous soils within the alpine zone and is commonly found beneath the edges of groves. It is distributed in America [43], Austria [74], Canada [44], France [46], Germany [47], Greenland [48], Iberian Peninsula and Balearic Islands [49], Ireland [51], Poland [56], Russia [57], the Czech Republic [59], Ukraine [60].

Specimens examined: China, Yunnan Prov., Shangrila city, Birong Valley, 26°39′04.26″ N, 99°44′13.77″ E, Alt. 3047 m, 15 July 2023, L. S. Wang et al., 23-75281(b); Dali city, Cangshan Geopark, 25°41′42.09″ N, 100°06′29.97″ E, Alt. 3092 m, 19 May 2024, L. S. Wang et al., 24-76095, 24-76098 (a), 24-76097, 24-76099. Xizang Prov., Linzhi, Nyingchi Sexiula Mountain G318 Roadside, 28 June 2021, CMY-42. Sichuan Prov., Liangshan Yi Autonomous Prefecture, Muli Co., 27°43′30.93″ N, 101°14′11.34″ E, Alt. 3070 m, 11 September 2021, X. Y. Wang et al., 21-70424; Jiulong Co., Wuxuhai Lake, 29°09′06.00″ N, 101°24′42.99″ E, Alt. 3722 m, 9 June 2023, L. S. Wang et al., 23-75118, 23-75119, 23-75121, 23-75122; Heishui Co., 32°13′57.72″ N, 102°36′21.75″ E, Alt. 3956 m, 4 September 2020, X. Y. Wang et al., XY20-399. Gansu Prov., Luqu Co., 34°08′23.63″ N, 102°11′55.61″ E, Alt. 3584 m, 9 July 2022, X. Y. Wang et al., XY22-1164. Austria, Salzburg, Lungau Eastern Alps, Niedere Tauern, Schladminger Tauern, Weißpriachtal NW of the village Mariapfarr, a short distance from Lahnbrücke, 47°13′18″ N, 13°39′24″ E, Alt. 1280 m, 3 September 2019, Y. Y. Zhang, ZYY-126.

**Figure 12:**
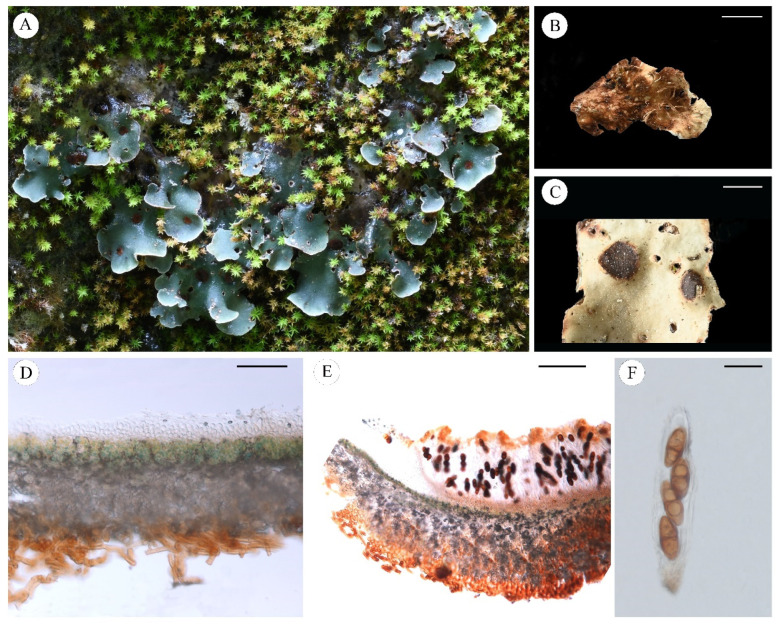
*Pseudosolorina tenuior* (L. S. Wang et al. 23-75283 KUN); (**A**), habit of *P*. *tenuior*; (**B**), white lower surface with brownish tomentum and rhizines; (**C**), apothecia; (**D**), transversal sections of the thallus; (**E**), transversal sections of the apothecium; (**F**), ascus. Scale bars: 5 mm (**B**); 2 mm (**C**); 100 µm (**D**); 200 µm (**E**); 40 µm (**F**).

***Pseudosolorina tenuior*** T. Zheng and Li S. Wang, sp. nov. **Figure 12**

Fungal Names: FN 572278

Type: China, Yunnan Prov., Shangrila city, Birong Valley, 26°39′05.36″ N, 99°44′15.57″ E, Alt. 3406 m, 15 July 2023, L. S. Wang et al., 23-75283 (Holotype KUN)

Etymology: Referring to its thin thallus and photobiont layer.

Diagnosis: This species contains a single photobiont layer with both cyanobacterial and chlorophyte cells. It has the same four secondary metabolites as *P. simensis*, but *P. simensis* has a thicker photobiont layer: more than 70 μm in thallus and more than 60 μm in apothecia. *P. tenuior* has a thinner photobiont layer: less than 60 μm in thallus and less than 30 μm in apothecia.

Description: Thallus: large foliose, fragile, dorsiventral, heteromerous, lobate, 3–7 cm in diam.; Lobes: rounded, 3–18 mm; Upper surface: green-gray when wet, gray-brown when dry, rare pruinose on the margin; Upper cortex: paraplectenchymatous, colorless, 30–80 μm thick; Medulla: 200–270 μm thick; Photobiont: *Asterochloris* and *Nostoc*, in a single photobiont layer, measured 20–60 μm, photobiont layer in apothecium measuring 12–30 µm; Lower cortex: lacking; Lower surface: pale to brown, indistinctly veined, tomentose, without cephalodia, with clusters of simple or branched rhizines; Apothecia: large, rounded, irregularly scattered, sunk in depressions in the upper surface, red-brown to black, slightly concave, thalline margin absent; Asci: clavate, *Peltigera*-type, 4-spored; Ascospores: red-brown or dark brown, 1-septate with a median constriction, ellipsoid to fusiform, wall non-uniformly thickened, surface ornamented with broad reticulating ridges and irregular lacunae, dimensions: 40–55 × 18–23 µm.

Chemistry: Gyrophoric acid, methyl gyrophorate, tenuiorin, and 2′-*O*-methyltenuiorin (detected by TLC and HPLC).

Ecology and distribution: It is distributed in high-altitude areas and is mainly found in calcareous soil on stone, usually growing together with or on bryophyte. This species is known from Yunnan and Sichuan Provinces of China.

Note: This species is similar to *Pseudosolorina simensis* in thallus morphology, appearance of photobiont layer, and chemical characteristics, but DNA sequence data support their description as separate species. They differ in the thickness of the photobiont layer both in thallus and in apothecium: the photobiont layer of *P. tenuior* is 20–60 μm in thallus and 12–30 µm in apothecium, but the photobiont layer of *P. simensis* is 70–150 μm in thallus and 60–100 µm in apothecium.

Specimens examined: China, Sichuan Prov., Liangshan Yi Autonomous Prefecture, Muli Co., 27°43′30.80″ N, 101°14′10.55″ E, Alt. 2993 m, 11 September 2021, X. Y. Wang et al., XY21-270; Yunnan Prov., Shangrila city, Birong Valley, 26°39′05.36″ N, 99°44′15.57″ E, Alt. 3406 m, 15 July 2023, L. S. Wang et al., 23-75283; Shangrila city, Birong Valley, 26°37′50.66″ N, 99°43′20.06″ E, Alt. 3080 m, 15 July 2023, L. S. Wang et al., 23-75759.

## 4. Conclusions

The revised circumscription of *Solorina* now comprises two distinct species, *S. crocea* and *S. crocoides*, which are distinguished by the unique characteristics of their thallus, photobiont layers, and chemistry. Specifically, *S. crocea* can be identified by its smooth upper surface with apothecia and the presence of two photobiont layers. *S. crocoides* is characterized by dark granules along the thallus margin and only a single photobiont layer containing both cyanobacteria and chlorophyte cells. No apothecia have been observed. These two species share similar secondary metabolites, but *S. crocea* contains gyrophoric acid, which is absent in *S. crocoides*.

Species within the new genus *Pseudosolorina* can be categorized into three distinct groups based on characteristics, including color and size of their thalli and formation of the photobiont layer.

The first group, *P. hepatizon*, *P. simensis*, and *P. tenuior* comprises species with greyish-brown upper surfaces and a single photobiont layer containing both cyanobacteria and chlorophytes. *P. hepatizon* differs from the other two species by its lack of secondary metabolites. *P. simensis* and *P. tenuior* both contain gyrophoric acid, methyl gyrophorate, tenuiorin, and 2′-*O*-methyltenuiorin. *P. simensis* differs from *P. tenuior* by the thickness of its photobiont layer (70–150 μm) relative to *P. tenuior* (20–60 μm). The second group features species with green upper surfaces, relatively large thalli (2–5 cm in diam.), and possession of a single green photobiont layer containing solely chlorophytes. This group consists of two species: *P. parmigera* and *P. saccata*. *P. parmigera* exhibits a more pruinose upper surface without external cephalodia, whereas *P. saccata* is rarely pruinose and exhibits external cephalodia on the upper surface. The third group comprises three species with relatively small thalli (<2 cm in diam.). Thalli of *P. spongiosa* form only a small circle around the apothecium. The most significant distinction between the three species is their number of ascospores; specimens of *P. bispora* have two spores, *P. bispora* var. *monospora* has one large spore, and *P. spongiosa* has four spores.

Key to species of *Solorina* and *Pseudosolorina*1a. Lower surface bright orange, containing solorinic acid and averantin21b. Lower surface white or pale brown, without solorinic acid and averantin32a. Thallus with smooth margin, contains two separate photobiont layers
*S. crocea*
2b. Thallus with sorediate margin, contains a single photobiont layer
*S. crocoides*
3a. Upper surface gray-brown, with single photobiont layer containing cyanobacteria and chlorophytes43b. Upper surface apple green, with single photobiont layer containing chlorophytes64a. Lacking secondary metabolites
*P. hepatizon*
4b. Containing secondary metabolites55a. Photobiont layer 20–60 µm in thallus and 12–40 µm in apothecium
*P. tenuior*
5b. Photobiont layer 70–150 µm in thallus and 60–100 µm in apothecium
*P. simensis*
6a. Thallus well-developed, 2–6 cm in diam.76b. Thallus small, less than 2 cm in diam.87a. Upper surface pruinose, without external cephalodia
*P. parmigera*
7b. Upper surface rarely pruinose, with external cephalodia
*P. saccata*
8a. Thallus less than 0.7 cm in diam., asci 4-spored
*P. spongiosa*
8b. Thallus 1–2 cm in diam., asci (1-)2-spored99a. Asci 1-spored*P. bispora* var. *monospora*9b. Asci 2-spored
*P. bispora*


## Figures and Tables

**Table 1:** Taxon sampling and mycobiont data with corresponding GenBank numbers used in phylogenetic analyses of Peltigerales. Sequences newly acquired for *Solorina* and *Pseudosolorina* are indicated in bold.

Taxon	Voucher	Origin	GenBank No.		
			nrITS	nrLSU	mtSSU
***Collema flaccidum***	**2000120**	China	MW453229.1	EU982618.1	EU982578.1
*C. furfuraceum*	MA-16260	Spain	GQ396263.1	EU982608.1	EU982567.1
*Leptogium hibernicum*	Bjelland TB_RB_4	Norway	KX013723.1	KX013759.1	KX013684.1
*L. saturninum*	MA-Lichen 16024	France	DQ466043.1	EU982610.1	KX027281.1
*Lobaria amplissima*	1996 Stocker-Worgotter 1717	Norway: Hordaland	AF524923.1	AY340546.1	AY340500.1
*L. pulmonaria*	Spribille 39,224 (MSC)	USA: Alaska	MN483125.1	AF183934.2	AY340503.1
*Nephroma arcticum*	Goffinet 1310	USA	AF014109.1	DQ973040.1	DQ972989.1
*N. bellum*	133 LG 214	Canada	HQ455058.1	HQ394180.1	HQ423261.1
*N. expallidum*	CONN 9370	USA	HQ455059.1	HQ394183.1	HQ423263.1
*Pannaria conoplea*	O-L-196369	Norway	MK811685.1	AY424209.1	MT068515.1
*P. hookeri*	MJK3314	Argentina	MT755913.1	JX464118.1	MN634245.1
*P. rubiginosa*	LG:R1008	Reunion	KF704259.1	JX494269.1	JX494244.1
*Peltigera aphthosa*		Canada	U73492.1	AF286759.1	AY340515.1
*P. leucophlebia*	Goward 98-C	USA	AF158651.1	AF286753.1	AY124166.1
*P. malacea*		Canada	U73491.1	AF286756.1	MH792883.1
*Pseudosolorina bispora*	P6109	Canada: Nunavut	MT522618.1	MT573442.1	
*P. saccata*	KL18-0005	Korea	MK503160.1	MK506118.1	MK508904.1
*P. simensis* 1	L1045	France: Reunion Island	MT522616.1	MK517842.1	
*P. simensis* 2	L915	Rwanda	MT522617.1	MK517843.1	
*P. spongiosa*	08792 (HBG)	Austria: Schultz	MZ708642.1		KJ766495.1
*Psoroma cinnamomeum*	O-L-184538	Norway	MK811978.1	OL331762.1	OM103956.1
*P. hypnorum*	Passo 20 (BCRU 4914)	Argentina	EU885309.1	AY340565.1	AY340523.1
*Sinuicella denisonii*	OSC:Stone 10024	USA: Oregon	NR_182350.1	MT942675.1	MT942641.1
*Solorina crocea*	P6110	Finland	MT522619.1	MT573443.1	
*S. crocea* 2	AFTOL-ID 1619	USA: Durham		DQ973043.1	
*S. crocea* 3	McCune 23785	USA: Chicago		AF286824.1	
*Sticta beauvoisii*	Lg3303	USA: NC	KT281725.1	DQ986769.1	KT281681.1
*S. weigelii*	1997 Stenroos 4816 (TUR)	Guyana	AF524905.1	EU558794.1	EU558865.1
*Vahliella leucophaea*	Ekman 3202 (BG)	Norway	AF429266.1	JX464125.1	HQ268596.1
***Pseudosolorina bispora***	**20-66618 (KUN)**	**China: Sichuan**	**PQ627847**	**PQ623371**	**PQ623429**
***P. bispora***	**ZYY-46 (KUN)**	**Austria: Salzburg**	**PQ627812**		**PQ623428**
***P. bispora***	**24-76716 (KUN)**	**China: Yunnan**	**PQ627820**		**PQ623414**
***P. bispora*** var. ***monospora***	**20-67082 (KUN)**	**China: Qinghai**	**PQ627846**		**PQ623427**
***P. bispora*** var. ***monospora***	**XY20-2943 (KUN)**	**China: Qinghai**	**PQ627817**	**PQ623369**	**PQ623425**
***P. bispora*** var. ***monospora***	**XY20-828 (KUN)**	**China: Qinghai**	**PQ627816**	**PQ623370**	**PQ623426**
***P. hepatizon***	**17-55094 (KUN)**	**China: Yunnan**	**PQ627849**	**PQ623379**	**PQ623444**
***P. hepatizon***	**23-75286 (KUN)**	**China: Yunnan**	**PQ627835**	**PQ623378**	**PQ623443**
***P. parmigera***	**19-62636 (KUN)**	**China: Yunnan**	**PQ627850**		**PQ623437**
***P. parmigera***	**21-70423 (KUN)**	**China: Sichuan**	**PQ627843**		**PQ623435**
***P. parmigera***	**23-75739 (KUN)**	**China: Yunnan**	**PQ627831**	**PQ623374**	**PQ623436**
***P. parmigera***	**21-70363 (KUN)**	**China: Sichuan**	**PQ627845**		**PQ623434**
***P. parmigera***	**24-76096(KUN)**	**China: Yunnan**	**PQ627824**	**PQ623363**	**PQ623418**
***P. parmigera***	**XY21-272 (KUN)**	**China: Sichuan**	**PQ627819**		**PQ623413**
***P. saccata***	**23-75280 (KUN)**	**China: Yunnan**	**PQ627840**		
***P. saccata***	**23-75757 (KUN)**	**China: Yunnan**	**PQ627828**		
***P. saccata***	**23-75281a (KUN)**	**China: Yunnan**	**PQ627839**	**PQ623365**	**PQ623421**
***P. saccata***	**XY23-383 (KUN)**	**China: Yunnan**	**PQ627813**	**PQ623372**	**PQ623430**
***P. saccata***	**23-75282 (KUN)**	**China: Yunnan**	**PQ627837**		**PQ623433**
***P. saccata***	**23-75755 (KUN)**	**China: Yunnan**	**PQ627830**	**PQ623373**	**PQ623432**
***P. saccata***	**23-75756 (KUN)**	**China: Yunnan**	**PQ627829**		**PQ623431**
***P. saccata***	**24-76098b (KUN)**	**China: Yunnan**	**PQ627822**		**PQ623416**
***P. simensis***	**21-70422 (KUN)**	**China: Sichuan**	**PQ627844**		
***P. simensis***	**XY21-274 (KUN)**	**China: Sichuan**	**PQ627815**		**PQ623438**
***P. simensis***	**23-75761 (KUN)**	**China: Yunnan**	**PQ627826**	**PQ623375**	**PQ623439**
***P. spongiosa***	**21-70424 (KUN)**	**China: Sichuan**	**PQ627842**		**PQ623424**
***P. spongiosa***	**23-75118 (KUN)**	**China: Sichuan**	**PQ627841**	**PQ623368**	
***P. spongiosa***	**23-75281b (KUN)**	**China: Yunnan**	**PQ627838**	**PQ623364**	**PQ623420**
***P. spongiosa***	**24-76095 (KUN)**	**China: Yunnan**	**PQ627825**		**PQ623419**
***P. spongiosa***	**24-76098a (KUN)**	**China: Yunnan**	**PQ627823**		**PQ623417**
***P. spongiosa***	**CMY-42 (KUN)**	**China: Xizang**	**PQ627818**	**PQ623367**	**PQ623423**
***P. spongiosa***	**XY22-1164 (KUN)**	**China: Gansu**	**PQ627814**	**PQ623366**	**PQ623422**
***P. spongiosa***	**24-76099 (KUN)**	**China: Yunnan**	**PQ627821**	**PQ623359**	**PQ623415**
***P. tenuior***	**23-75283 (KUN)**	**China: Yunnan**	**PQ627836**		**PQ623442**
***P. tenuior***	**XY21-270 (KUN)**	**China: Sichuan**	**PQ627848**	**PQ623376**	**PQ623440**
***P. tenuior***	**23-75759 (KUN)**	**China: Yunnan**	**PQ627827**	**PQ623377**	**PQ623441**
***Solorina crocoides***	**23-75431 (KUN)**	**China: Xizang**	**PQ627834**	**PQ623362**	
***S. crocoides***	**23-75450 (KUN)**	**China: Xizang**	**PQ627832**	**PQ623360**	
***S. crocoides***	**23-75449 (KUN)**	**China: Xizang**	**PQ627833**	**PQ623361**	

**Table 2:** The photobionts sequenced from *Solorina* and *Pseudosolorina*, with corresponding GenBank numbers. The horizontal line signifies that the information is not yet available.

Host		Chlorophyta		Cyanobacteria	
Taxon	Voucher	Taxon	GenBank No.	Taxon	GenBank No.
			ITS		16S RNA
*Pseudosolorina bispora*	24-76716	*Coccomyxa* sp.	PV085834	*Nostoc* sp.	PV083155
*Pseudosolorina bispora* var. *monospora*	20-67082	*Coccomyxa* sp.	PV085836	*Nostoc* sp.	PV083158
*Pseudosolorina hepatizon*	17-55094	*Asterochloris* sp.	PV085839	*Nostoc* sp.	PV083157
*Pseudosolorina spongiosa*	24-76095	*Coccomyxa* sp.	PV085835	*–*	*–*
*Pseudosolorina parmigera*	24-76096	*Coccomyxa* sp.	PV085837	*–*	*–*
*Pseudosolorina saccata*	XY23-383	*Coccomyxa* sp.	PV085838	*–*	*–*
*Pseudosolorina simensis*	23-75761	*–*	*–*	*Nostoc* sp.	PV083160
*Pseudosolorina tenuior*	23-75283	*–*	*–*	*Nostoc* sp.	PV083159
*Solorina crocoides*	23-75450	*Asterochloris* sp.	PV085840	*Nostoc* sp.	PV083156

## Data Availability

The original contributions presented in the study are included in the article, further inquiries can be directed to the corresponding author.
